# Letter from “J”

**Published:** 1863-06

**Authors:** 


MR. EDITOR:

Not having been solicited to write for the Register or to "take the wind out of any body's sails," nevertheless I infer the privilege granted.

The author of the article on "Fang Fillings," claims to be an "Enthusiast" and to have his hobbies, consequently he must be endulged in the exclaimation, "Eureka."

"I can't see it," his method is not simple, and we deny its effectiveness. Alveolar abscesses can *always* be cured in patients of good recuperative powers without the exclusive aid of fang fillings. The author uses "Arsenic" ground in a mineral mortar for two hours, and minutely divided with equal parts—by weight—of sugar of milk, and converted into a paste with creosote. (This latter article the "author" has often denied the use of.) The arsenic, he claims, is dissolved readily and absorbed, acting promptly." I don't doubt it, and have in my mind now one case of "prompt action" in his practice which is not mentioned in his "astute" article. The broach is called into action to aid the arsenic after it is claimed to have done its work, and after he has repudiated the broach as incompetent; this is consistency in a horn. According to his own writing, the correspondent does not remove the destroyed nerve after severing its connection; but at the expiration of a week or ten days and after frequent washing out with tepid water, he removes "very readily" the dead nerve. "If the cavity be irregular or too small, enlarge it with a reamer made in the form of a broach." In how many cases can the author accomplish this tremendous *theory?* In about one in every one hundred, or very rarely in a convenient incisor. I deny its possibility as a general rule, and don't believe the writer himself has implicit confidence in it. Piano wire is a good thing in its place, but cannot possibly be converted into drills for enlarging nerve canals.

Now comes the "tug of war." "The first piece of gold must go clear to the appex of the fang," and be there consolidated. Does it do it? I grant it will go just as readily as the operator can put it there; but both are in the main utter impossibilities. A broach is used for a plugger—bully for the broach. How solid my friend can you make a fang filling with a broach? This must be an enthusiastic idea. After this splendidly theoretical job is finished, the author fears no evil "Ceterus" paribus. This ceterus paribus comes in at the right time, and is very fortunately applied. "Other things being equal" almost any operation would prove a success; but other things are not always equal, and fact says, in the majority of cases these gold fang fillings are failures. I do not suppose it would be difficult for the "author" to find failures in his enthusiastic practice.

The part of his article which treats of teeth with dead nerves is more rational, but as it was intended for the profession terms suitable should have been introduced instead of talking about "scraping the jaw bone."

Now laying aside all levity, the practice referred to is simply impossible, splendid in theory, but entirely at fault in practice. Let the "author" give us the truth of the matter and the reasonable approximation to his theory, and we will say he writes well, and possibly may meet with an occasional success in his "enthusiastic hobbies." Such articles in my humble opinion are calculated to do a great deal of harm among some practitioners, but others will give them all the attention they merit. Dr. Clark, of New Orleans, was one of the pioneers of this *theory,* but he was candid enough to concede it was theory, and not the ultimatum or "Eureka." Having been engaged in the practice of Dentistry, at least as long as the author, and having had access to all improvements, and having investigated all new theories, I am prepared to give to the article all the merit there is in it as a tolerably well written explanation of a "hobby."

Some dentists have had good classical educations, and Theorists should always quote correctly. Let us have facts and successes in modes of practice, truthfully detailed, and let hobbies be discarded. If time and space would permit, I should like to devote a few lines to an "Enthusiast" in Dayton, Ohio, concerning his theory of *prostituting* mechanical dentistry, but as he may be classed among the "babbling brooks," we will let him slide for the present. J.

